# Anatomical Considerations and Plastic Surgery Reconstruction Options of Sacral Chordoma Resection

**DOI:** 10.7759/cureus.37965

**Published:** 2023-04-22

**Authors:** Parthena Deskoulidi, Spyros D Stavrianos, Dimitris Mastorakos, Vasileios A Kontogeorgakos, Olga Savvidou, Dimosthenis Chrysikos, Alexandros Samolis, Nikolaos Pappas, Theodore Troupis, Panayiotis J Papagelopoulos

**Affiliations:** 1 Department of Plastic and Reconstructive Surgery, KAT Hospital, Athens, GRC; 2 Department of Plastic and Reconstructive Surgery, Saint Savvas Hospital, Athens, GRC; 3 Department of Plastic and Reconstructive Surgery, Athens Breast Clinic, Athens, GRC; 4 First Department of Orthopedic Surgery, National and Kapodistrian University of Athens, Medical School, Athens, GRC; 5 Department of Orthopedic Surgery, Attikon University General Hospital, Athens, GRC; 6 Department of Anatomy, National and Kapodistrian University of Athens, Medical School, Athens, GRC

**Keywords:** sacral tumors, radiotherapy (rt), advancement flap, partial sacrectomy, chordomas

## Abstract

Introduction

Chordomas are slow-growing malignant bone tumors arising from remnant embryonic notochord cells with predilection for the sacrum. They rarely metastasize, and early surgical resection with clear margins is the treatment of choice followed by plastic surgery reconstruction supplemented with adjuvant radiotherapy based on the local treatment protocol or in cases with a contaminated surgical field.

Aim

The aim of the present study is to present our experience in surgical management of sacral chordomas and propose a surgical reconstruction algorithm considering anatomical parameters after partial or total sacrectomy.

Materials and methods

Twenty-seven patients with sacral chordomas were treated in our Orthopaedic Surgery Department between January 1997 and September 2022, and 10 of them had plastic surgery reconstruction. Patients were divided into groups based on the type of sacrectomy, sacrum anatomical vascular or neural variations, partial or total, and the type of soft tissue reconstruction. The postoperative complications and the functional outcomes in each patient were assessed.

Results

Bilateral gluteal advancement flaps or gluteal perforator flaps are the first choice in patients with partial sacrectomy, intact gluteal vessels, and without preoperative radiotherapy followed by transpelvic vertical rectus abdominis myocutaneous flap or free flaps in those patients with near total sacrectomy and preoperative radiation therapy.

Conclusion

There are four reliable options for patients after sacral chordoma resection: direct closure, bilateral gluteal advancement flaps, transpelvic vertical rectus abdominis myocutaneous flaps, and free flaps. Each time, tumor-free margins and a good reconstructive plan according to the defect and patient characteristics are mandatory.

## Introduction

Chordomas which are also called notochordal sarcomas are uncommon malignant bone tumors that originate from the notochord, a tissue that comes from the mesoderm which is crucial for correct embryonic patterning. The remnants of embryonic notochord cells which do not disappear after birth can turn into chordomas and commonly involve the anatomical region of the axial skeleton (sacrococcygeal, spinal, or spheno-occipital regions). They account for 1.4% of all primary malignant bone tumors, and just 0.2% of spinal tumors, working out to 300 patients who are diagnosed with chordoma each year in the United States, and about 700 in all of Europe. At any given time, fewer than 1 in 100,000 people are living with chordomas [1] A German doctor, Dr. Rudolf Virchow initially described chordoma histologically in 1846 who described it as a soft, transparent, and multi-lobulated with an underlying myxoid structure that appeared “slimy” and later posited that these lesions were cartilaginous in nature and resulted from hydropic degeneration of the spheno-occipital junction with concomitant softening of the cartilage matrix [2]. Notochord-derived lesions are categorized in contemporary descriptions as benign notochordal cell tumors and malignant chordomas. Dr. Edwin Klebs of Berlin provided the first clinical description in 1864 [3]. He presented an instance of a possible spheno-occipital junction chordoma. The removal of a presacral chordoma in a 46-year-old female patient was originally documented by Feldmann in 1910, but long-term follow-up was not reported. Several authors published reviews of the literature, tumor localization, patient epidemiology, clinical presentation, surgical excision, management of the defects, surgical reconstruction, and the quality of life of the patients after excision and reconstruction and oncological treatment. In the 1970s, Stener and Gunterberg first introduced the idea of wide en bloc surgical resection for the treatment of sacral tumors [4]. Since then, en bloc excision has remained the central idea in the surgical management and more aggressive surgery and wider surgical margins improved the local control of the disease recurrence for sacrum chordomas. The average age at diagnosis is 54.1 years with a male-to-female ratio of 2:1 and younger patients faring better than older ones. Patients with sacral chordomas most commonly present with local lower back or flank pain which deteriorate on sitting and up to a third of these patients have associated urinary tract infections, constipation, or symptoms of disc herniation. Patients with advanced chordomas have a poor prognosis and suffer from multiple locoregional relapses and the reported incidence of metastases in chordoma is between 3-48% to the lungs, bones, lymph nodes, and liver. The treatment of chordomas most often involves surgical resection followed by radiotherapy or excision and reconstruction followed by radiotherapy after one month to avoid wound healing problems. Cytotoxic chemotherapy or other systemic therapies have not been proven to be effective for chordomas. Several molecularly targeted therapies have been proposed to be effective, but the literature is poor [1]. The aim of this article is to present and evaluate our results in surgical management of sacral chordomas and propose a surgical reconstruction algorithm after partial or total sacrectomy taking into account anatomical parameters of sacrum or vascular and neural variations.

## Materials and methods

The medical records of 27 patients who underwent partial or total sacrectomy and reconstruction or no reconstruction between January 1997 and September 2022 (18 men and 9 women; Table 1) with a mean age of 63 years were reviewed at the First Department of Orthopaedic Surgery of Athens Medical School. Ethics approval has been obtained for the study from the Ethics Committee of Attikon University Hospital with the approval number EBD214/30-03-2022. Our cases were managed by a well-prepared multidisciplinary team that consisted of orthopedic surgeons, plastic surgeons, urologists, radiologists, general surgeons, pathologists, radiotherapists, and at least one medical oncologist [5]. The size of sacrum chordomas, the type of sacrectomy, the type of reconstruction, the history of radiation therapy or not, postoperative complications, and restoration of function were determined in each patient with a mean length of follow-up ranging from 1 to 5 years. Sacrectomy was typically performed by orthopaedic surgeons. Written informed consent from all patients was obtained preoperatively. Seventeen patients with smaller sacral chordomas below S2 or S3, not protruding anteriorly and without preoperative radiation, were operated in the prone position. The goal of surgery is to achieve negative microscopic margins which is the most important prognostic factor for recurrence or not. Around the prior biopsy incision, a fusiform incision was made from the lower back to the coccyx, and flaps were created to the sacral margins. At each side's sacral border, the gluteus maximus muscle fibers were separated. The sacral lateral edge was medially separated from the piriformis muscle. The front surface of the coccyx, sacrum, and rectum were dissected from the rectum until a tumor was palpable between the sacrum and rectum. The bone was revealed posteriorly at a point close to where its intended division would have occurred. The division would be below the S3 level and unlikely to harm the proximal pudendal nerve roots and result in incontinence if the exposed posterior and anterior surface of the sacrum could be divided in a straight line from edge to edge above the tumor. If the rectum is not resected after specimen removal, it bulges posteriorly [6]. The gluteal fascia cannot be approximated because a significant section of it was removed. Suction drains are placed in the wound, exiting through the skin close to the upper corner of the incision. Scarpa fascia and the dermis are approximated with interrupted absorbable 2-0 Vicryl sutures.

Ten patients with larger sacral chordomas had partial or total sacrectomy followed by plastic surgery reconstruction. Gluteal flaps were performed for five patients with partial sacrectomy [7]. Transpelvic vertical rectus abdominis myocutaneous (VRAM) flaps were performed for five patients with total sacrectomy [8]. Total sacrectomy was performed with a combined transabdominal and a posterior sacral route approach for aggressive chordomas below L5. A longitudinal midline incision from 5 cm above the umbilicus to the lower abdomen was used for the anterior approach. We located both ureters using a transperitoneal approach, freed them of the tumor, and tied off both internal iliac arteries and veins as well as the middle sacral vessels. The rectum was mobilized off the tumor if feasible. The L5-S1 disc was then partially exposed and removed.

In order to release the presacral fascia, sacrotuberous ligaments, sacrospinous ligaments, and piriformis, the patient was then rolled to the prone position and dissected from the posterior approach just lateral to the sacrum. Moreover, the lower sacral nerve roots were severed and tied. The most caudal nerve roots to be preserved were identified during laminectomy using information from the preoperative MRI. These roots were guarded and could be followed all the way to the sciatic nerves. Underneath the surviving nerve roots, the dural sac was separated and ligated. To link to the L5-S1 disc from the anterior approach surgery, the L5-S1 disc was recognized and divided. The iliac wings were then made visible. Depending on the planned type of sacral resection, the vertical osteotomies of the sacrum were carried out utilizing a number of precise osteotomes at the proper locations. The sacral tumor specimen was removed entirely after being dislodged from its surrounds.

In most of the cases, in order to achieve the desired resection margins, nerve roots will need to be sacrificed, and the functional outcome will depend on the involved roots. Partial sacrectomy often leads to sacrifice of the roots distal to S3 with a minimal deficit of a variable reduction in perineal sensation and sexual function. Total sacrectomy in which S1 roots are removed leads to loss of sphincter control, sexual function, and plantar flexion. We try to preserve at least one S3 root to avoid such problems.

Plastic surgery reconstruction and surgical anatomy

A total of 10 patients underwent plastic surgery reconstruction following partial or total sacrectomies, five patients with gluteal flaps and five patients required transpelvic vertical rectus abdominis myocutaneous (VRAM) flaps.

The bilateral gluteal rotational or advancement flaps was the first surgical option for the patients after removal of the sacrum chordoma for sacral defects of 12*20 cm to 15*21 cm.

We first marked the location of the superior gluteal artery which is one-third of the distance between the posterior superior iliac spine and the greater trochanter and inferior gluteal artery which is half the distance between the posterior superior iliac spine and the ischial tuberosity. A handheld Doppler was used to confirm the location. We marked and elevated a larger flap than needed by 3-4 cm. The flap design incorporated the superior gluteal artery and its perforators and or the inferior gluteal artery and perforators, depending on size needs.

The flap is designed in a triangular shape for a v-y advancement flap. We incised the skin overlying the gluteus maximus muscle beveling out to maintain the perforators and adequate blood supply to the flap. We dissected the flap from lateral to medial subcutaneously a few cm and divided the muscle from the origin at the sacrum. We took care not to injure the superior or gluteal artery vessels. Once the skin paddle was fully divided subcutaneously and submuscularly to the proximal sacrum part, the tissue was advanced easily medially from both sides. We sutured the flap gluteus maximus to the contralateral gluteus muscle with absorbable 2-0 Vicryl sutures. We placed drains, one on each side, and skin closure was performed with 3-0 monocryl sutures (Ethicon Plus Antibacterial, Ethicon, Inc., New Jersey, USA) and interrupted 3-0 nylon sutures (Atlas Medical, Cumberland, Rhode Island, USA).

For the myocutaneous rotation flap, we dissected the flap completely submuscular and took care not to undermine any of the subcutaneous tissue off of the muscle. The gluteus maximus muscle was detached at the lateral and inferior borders and elevated in a superomedial direction in the areolar plane above the gluteus medius. After identification and protection of the superior and/or inferior gluteal artery, we detached the gluteus maximus from its origin at the posterior superior iliac spine and rotated the flap over the sacral defect. The transpelvic VRAM flap was performed in five patients with total sacrectomy defects after chordoma resection.

The rectus abdominis muscle originates from the 6th, 7th, and 8th costal cartilages and inserts on the pubic bone. It measures approximately 6 cm in width at its origin and 3 cm in width at the insertion. It is a Type III Mathes/Nahai muscle flap due to its dual blood supply, superiorly from the superior epigastric artery, a branch of the internal mammary artery and inferiorly from the deep inferior epigastric artery, a branch of the external iliac artery. The deep inferior epigastric artery originates from the external iliac vessels at a point between the inguinal ligament to about 6 cm above it. The vessel enters the posterior rectus sheath at about the level of the ASIS and the arcuate line (4 to 6 cm above the pubic bone). This artery usually splits into a lateral and medial branch that supplies the lateral and medial row of myocutaneous perforators. There are three distinct branching patterns of the deep inferior epigastric artery above the arcuate line according to Moon and Taylor (type 1: the vessel ascends a single intramuscular artery, type 2: the deep inferior epigastric artery is divided at the arcuate line into two major intramuscular perforators, and type 3: trifurcation of the deep inferior epigastric artery) [9]. Knowledge of perforator anatomy is important when attempting the fascial-sparing approach of VRAM harvest. The skin paddle for the VRAM flap is usually designed to include paraumbilical myocutaneous perforators.

Patients are placed in a lithotomy position and following the oncologic resection and colostomy, the plastic surgery team made a midline skin incision, and dissect down to the linea alba. Once the rectus sheath is identified, dissection was performed laterally, superficial to the rectus sheath, to locate periumbilical and medial row perforators to the skin paddle. After incision of the anterior rectus sheath 2 to 3 cm lateral to the midline but medial to the medial row perforators, we retracted the rectus abdominis muscle laterally to expose the posterior rectus sheath. We incised the posterior rectus sheath 2 cm lateral to the midline to gain intraperitoneal access and curved the incision back toward the linea alba at the arcuate line. Advancement and suturing of the posterior rectus sheath to the anterior rectus sheath were followed to protect the rectus abdominis muscle during the oncologic resection part of the procedure. The ostomy is usually passed through the contralateral rectus abdominis muscle. Design of the skin paddle was usually 5-7 cm in width and 12-15 cm in length (depending on the sacral defect) over the rectus abdominis muscle such that it will reach the defect once the muscle is transposed. We released the sutures that were previously placed between the anterior and posterior rectus sheath, and we completed the soft tissue dissection to isolate the skin paddle. Dissection of the flap from lateral to medial over the anterior rectus sheath and just lateral, to the lateral row perforators, was followed by incision of the anterior sheath just lateral to the lateral row perforators. Dissection of the lateral aspect of the anterior rectus sheath from the anterior surface of rectus abdominis muscle using a combination of finger dissection and electrocautery and carefully through areas of muscle inscriptions to prevent damage to the overlying rectus sheath or to the muscle itself. Dissection of the rectus muscle off the posterior rectus sheath and transection of the rectus muscle cranial to the skin paddle to ensure that the superior epigastric vascular pedicles are ligated. Rotation of the flap medially and transpose it into the abdomen and pelvis. We carefully inspected that the inferior epigastric artery and vein are dissected free from all soft tissue attachments to prevent compression and kinking of the pedicle. We left the rectus muscle attached at its origin on the pelvis to prevent tension on the pedicle. After the flap has been elevated and transposed into the pelvis, we placed two drains into the pelvic area and transposed the patient to the prone position. Then the VRAM flap was easily pulled through the pelvis without tension and the tip of the flap ended up posterior to the defect. We were careful to avoid twisting of the pedicle and de-epithelialized the skin paddle to the dimension necessary to reconstruct the skin defect. We used the rest of the epithelialized portion to close the sacrum skin defect with 2-0 Vicryl and 3-0 monocryl interrupted sutures and staples. We then turned again the patient supine to close the donor side of the abdomen and if it was not feasible due to tension, a component separation technique was performed. We sutured the rectus sheath using interrupted 2-0 PDS sutures (Ethicon Plus Antibacterial, Ethicon, Inc., New Jersey, USA) in figure-of-8 fashion and include both the anterior and posterior sheath in the closure superior to the arcuate line. We placed a drain superficial to rectus sheath and approximated the Scarpa fascia with interrupted 2-0 Vicryl sutures.

## Results

**Figure 1:**
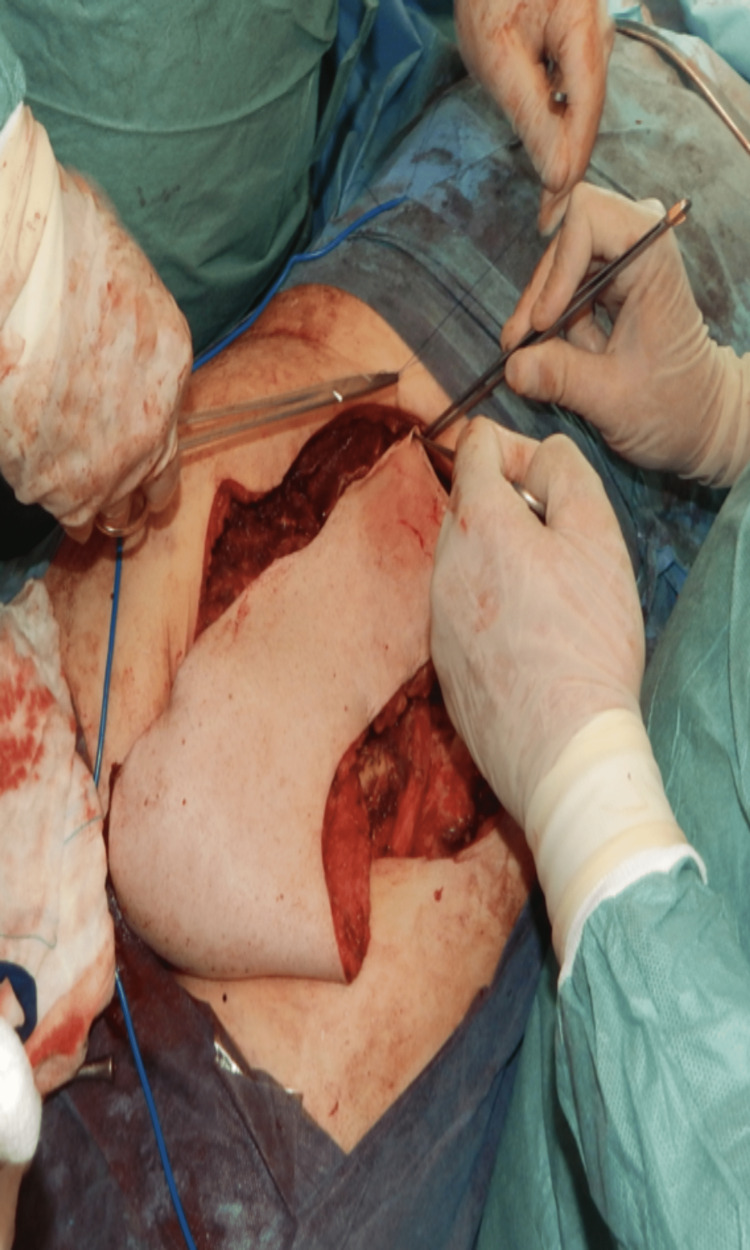
VRAM flap dissection VRAM: Vertical Rectus Abdominis myocutaneous flap

**Figure 2:**
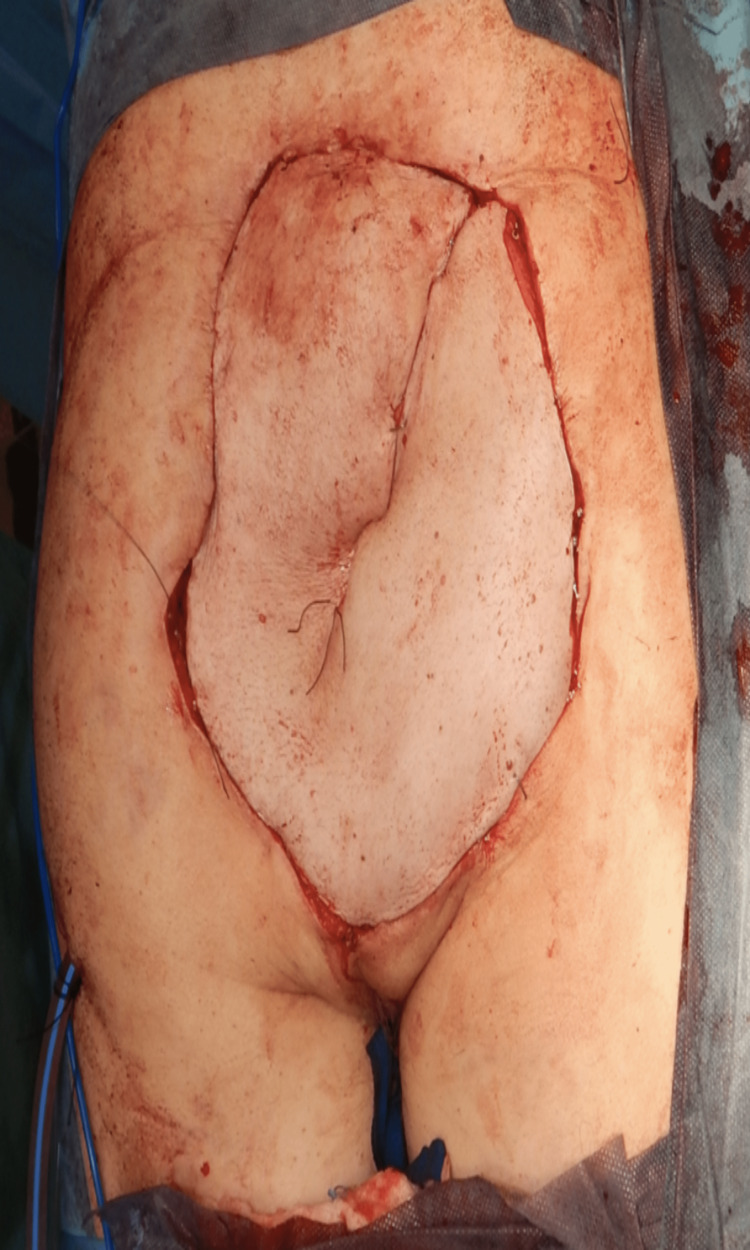
VRAM insetted through the pelvis to the defect area VRAM: Vertical rectus abdominis myocutaneous flap

**Figure 3:**
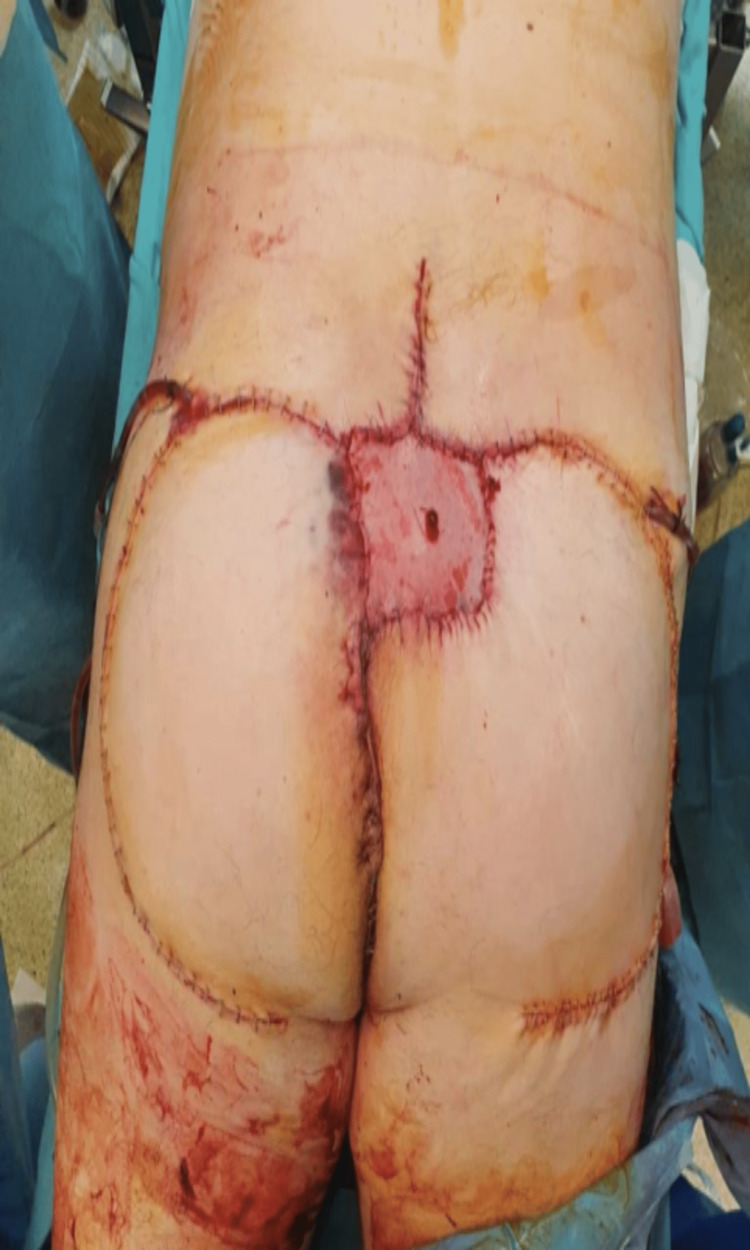
Deepithelialization of VRAM skin and final insetting of the flap VRAM: Vertical rectus abdominis myocutaneous flap

**Figure 4:**
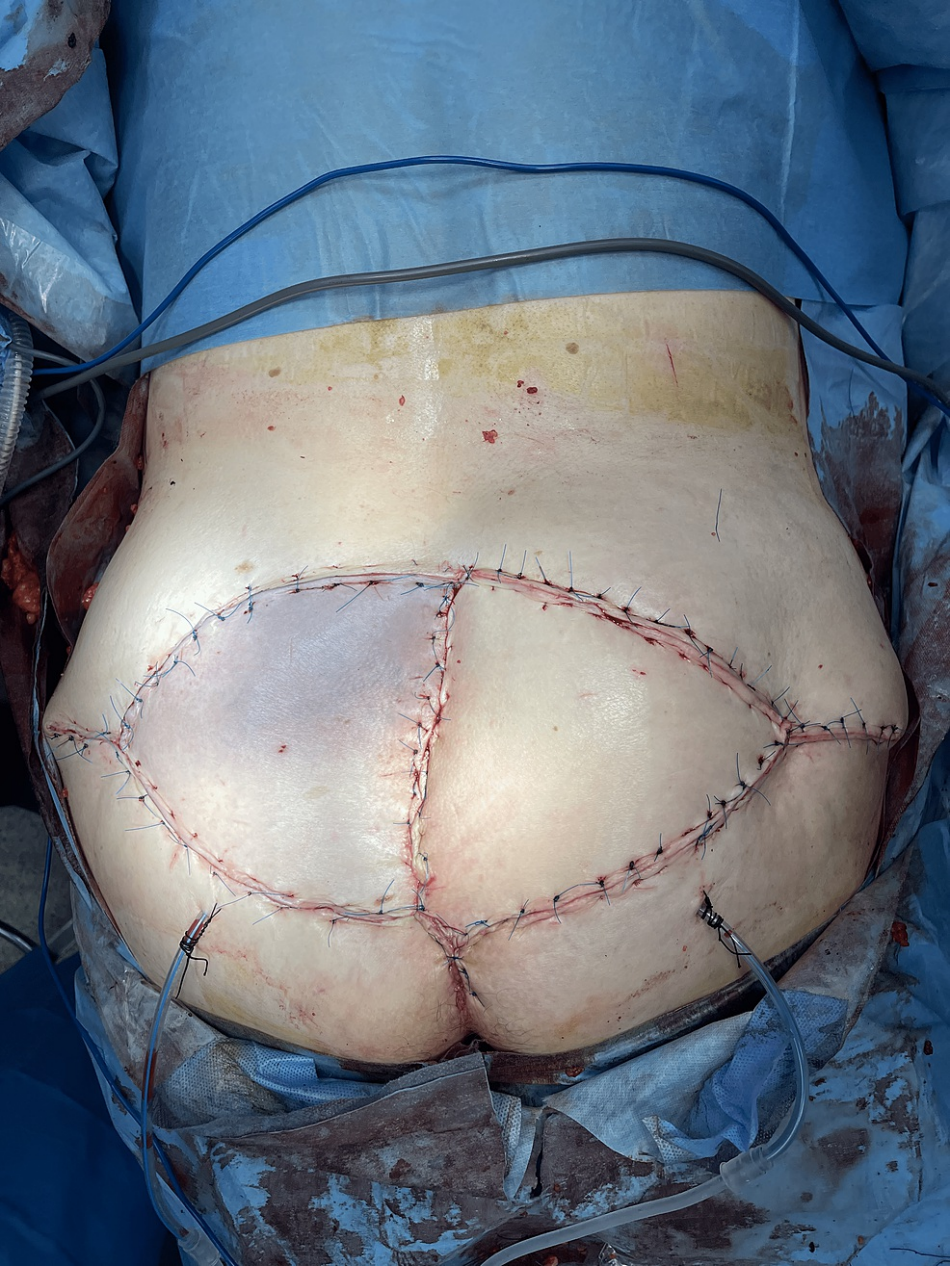
Bilateral gluteal advancement v-y flaps. Note the venous stasis to the L side

**Figure 5:**
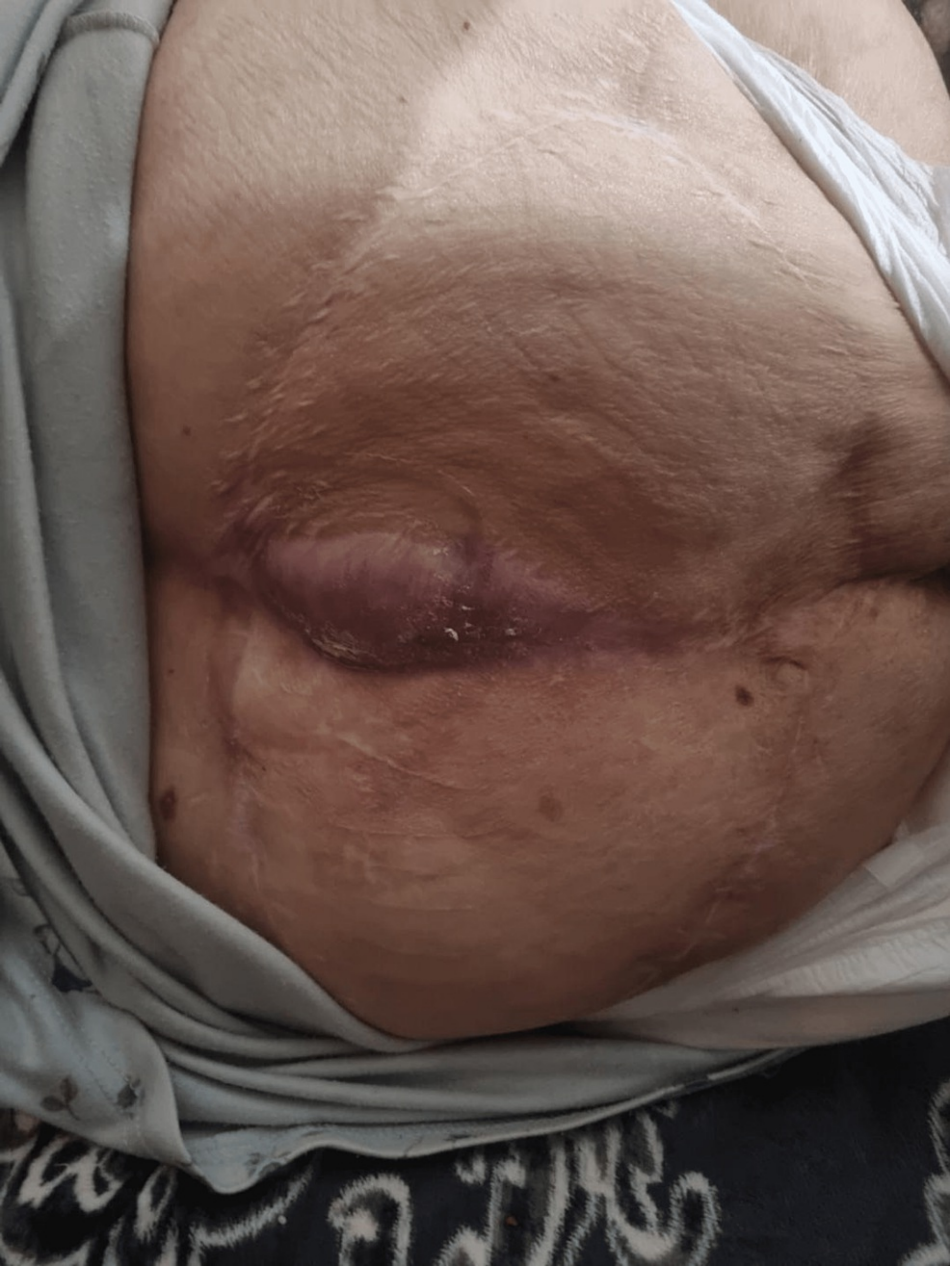
Postoperative result without recurrence after a year

**Figure 6:**
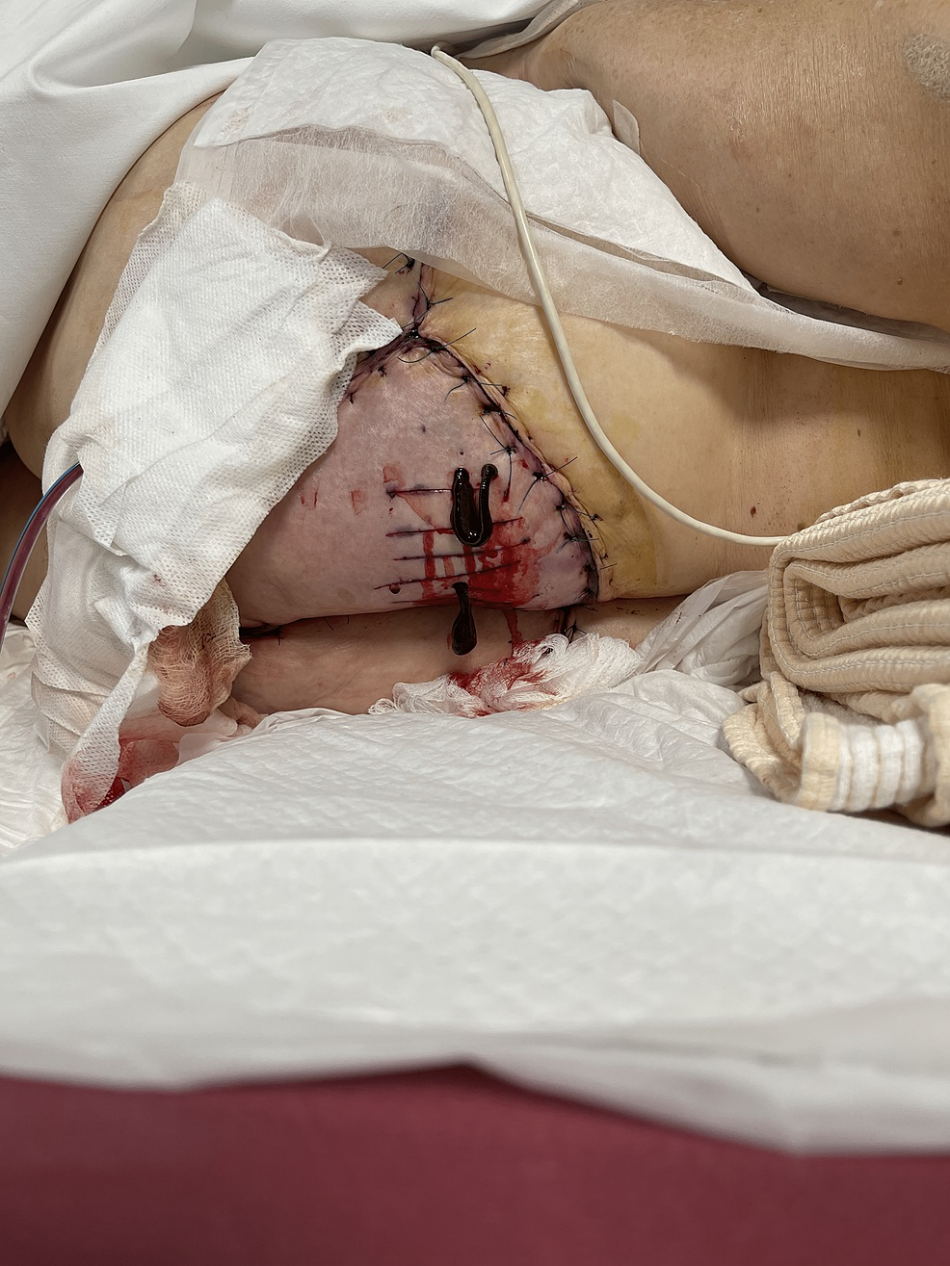
Medical leeches were used to encounter the venous stasis of the flap

The gluteal and VRAM flaps provided successful reconstruction for 9 out of 10 patients (Table 1 and Figures 1-3). There were three flap complications. Unfortunately, one patient after VRAM reconstruction developed a postoperative wound infection and a lower respiratory infection and died in the ICU clinic postoperatively due to sepsis (ASA classification score more than 3). One patient had partial necrosis of the VRAM flap which was managed conservatively with debridement and Vac therapy five days postoperatively and healed completely after three months. One patient with bilateral advancement gluteal v-y flaps had venous stasis to the one side of the flap which was managed with leech therapy treatment for 24 hours and completely healed (Figures 4-6). The mean follow-up time was 67 months. Three patients after flap reconstruction had chordoma recurrence and six without flap reconstruction, and they were managed with postoperative radiotherapy successfully.

**Table 1:** Treatment and outcome after sacrum chordoma resection in 27 patients VRAM: Vertical rectus abdominis myocutaneous flap

Patient	Chordoma size	Flap	Complications
1	3.5x3.5 cm	No	No
2	10x10 cm	VRAM	Wound infection
3	4x4.5 cm	No	Wound drainage
4	19x15 cm	No	No
5	13x12 cm	VRAM	No
6	15x15 cm	Gluteal	No
7	6X4 cm	No	Wound dehiscence
8	16x13 cm	Gluteal	Partial venous stasis
9	10x11 cm	Gluteal	No
10	25x10 cm	No	Wound dehiscence
11	8.5x9 cm	No	No
12	8x7 cm	No	Wound drainage
13	13x10 cm	No	No
14	7x6.5 cm	No	Wound dehiscence
15	20x20 cm	VRAM	Partial necrosis
16	12x9 cm	No	No
17	6x3.5 cm	No	No
18	16x8 cm	Gluteal	No
19	2.5x1.5 cm	No	No
20	9.5x8.5 cm	No	Wound drainage
21	10x9.5 cm	Gluteal	No
22	9x11 cm	Gluteal	No
23	4x5 cm	No	No
24	16x9 cm	VRAM	No
25	13x15 cm	No	No
26	5x7.5 cm	No	Wound dehiscence
27	6x8 cm	No	No

## Discussion

A wide variety of gluteal flaps are available according to the defect after partial or total sacrectomy due to chordoma excision and depend on the plastic surgeon’s ability, training, and creativity [10-12]. Local gluteal flaps offer the advantage of low donor site morbidity, are near the same surgical field, and can cover small to medium defects and sometimes larger defects if there is excess adipose tissue in the gluteal area. Furthermore, there is now a need to transfer the patient during the operation from the lithotomy position to prone position and back to the supine position. The only disadvantage is sometimes the venous congestion which was managed with leech therapy treatment. After the introduction of the perforator concept, confidence in anatomical knowledge and dissection especially for well-trained microsurgeons gives the ability in customized sacrum reconstruction [13]. For example, our half musculocutaneous and half fasciocutaneous v-y advancement gluteal flaps provide adequate coverage of the defect with the anterior portion of the gluteal muscles and better postoperative ambulatory function as the 2/3 of the flap is fasciocutaneous laterally.

The VRAM flap is used after total sacrectomies for larger defects and mainly in patients without previous abdominal surgery [14,15]. Three main disadvantages of the VRAM flap technique are the hernia risk of the abdominal donor side, the compression of the pedicle inside the tunnel which might compromise the blood flow of the flap, and the transfer of the patient from lithotomy to prone position something that increases the surgical overall time.

**Figure 7:**
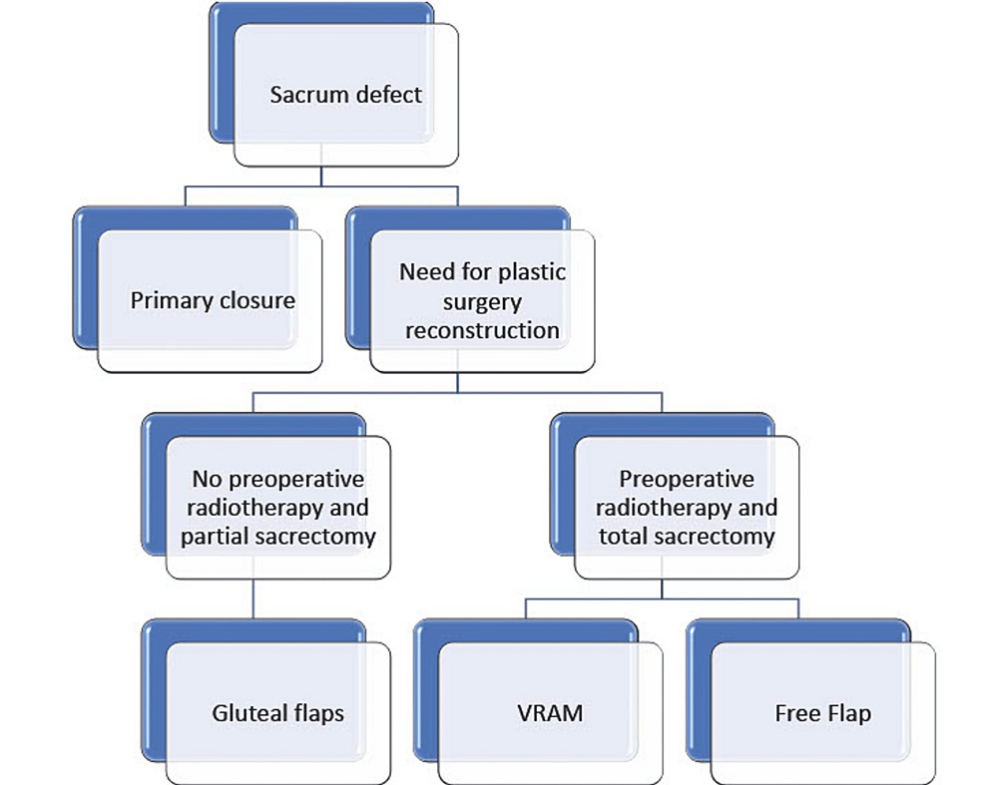
Our surgical algorithm for plastic surgery reconstruction after sacral chordoma resection VRAM: Vertical rectus abdominis myocutaneous flap

When local gluteal flaps or transpelvic VRAM cannot be performed due to previous radiotherapy or abdominal surgery, then a free flap would be the best option, but dissection of gluteal vessels or branches of gluteal vessels are tedious and complicated as recipient vessels. Our surgical algorithm for plastic surgery reconstruction after sacral chordoma resection is displayed in Figure 7.

This non-randomized, retrospective study's limited sample size is one of its limitations. However, the number of cases included in this study was comparable to the numbers reported in other published works in the literature, despite the study's limited sample size.

In our study, there was no connection between the postoperative ambulatory function and the technique of plastic surgery reconstruction. The degree of sacral resection and sacral nerve sacrifice seems to affect the functional result. The three patients who had chordoma recurrence after flap reconstruction had a clear but close margin below 1.5 mm and tumor contamination at the time of surgery, but the sacral nerve was preserved. Some deficits were observed during activities that required vigorous effort such as running and ascending stairs during the follow-up period. Postoperative MRI every four months is mandatory for the first two years and then every year to detect a recurrence in an early stage.

## Conclusions

To conclude, we present an adequate mapping of the gluteal vessels and perforators from the Ct, and a Doppler device is mandatory. They are included in the fasciocutaneous or musculocutaneous gluteal flaps which are our first option if the gluteal vessels are intact and there is no preoperative radiotherapy. If there is preoperative radiotherapy without previous abdominal surgery or a larger defect, it cannot be managed with gluteal flaps. The VRAM flap is our flap of choice.

The last option in sacrum reconstruction after chordoma excision due to the drawbacks of challenging dissection of the recipient gluteal vessels for microsurgical anastomosis, the need for additional surgical time due to multiple changing positions of the patient during the operation, and the risk of free flap failure might be effective if we do not choose the latissimus dorsi free flap. Each time, more aggressive surgery and wider surgical margins for the local control of disease recurrence followed by local gluteal advancement flaps or VRAM flaps are our options. Consideration needs to be given to the anatomy of the sacrum, the neural or vascular variations, and the best reconstructive options for sacrum chordoma patients according to their general medical conditions.

We have found this reconstruction plan depending on the defect, patient characteristics (medical conditions, adipose tissue in the buttock area, previous radiotherapy, or previous abdominal surgery), and good knowledge of surgical anatomy of the plastic surgeon particularly useful in sacrum reconstruction after chordoma resection.
